# Adaptation to environmental factors shapes the organization of regulatory regions in microbial communities

**DOI:** 10.1186/1471-2164-15-877

**Published:** 2014-10-08

**Authors:** Leyden Fernandez, Josep M Mercader, Mercè Planas-Fèlix, David Torrents

**Affiliations:** Joint IRB-BSC program on Computational Biology. BSC, Jordi Girona, 29, 08034 Barcelona, Spain; Institució Catalana de Recerca i Estudis Avançats (ICREA), Pg Luís Companys 23, Barcelona, 08010 USA

**Keywords:** Adaptation, Environment, Gene regulation, Metagenomes

## Abstract

**Background:**

It has been shown in a number of metagenomic studies that the addition and removal of specific genes have allowed microbiomes to adapt to specific environmental conditions by losing and gaining specific functions. But it is not known whether and how the regulation of gene expression also contributes to adaptation.

**Results:**

We have here characterized and analyzed the metaregulome of three different environments, as well as their impact in the adaptation to particular variable physico-chemical conditions. For this, we have developed a computational protocol to extract regulatory regions and their corresponding transcription factors binding sites directly from metagenomic reads and applied it to three well known environments: Acid Mine, Whale Fall, and Waseca Farm. Taking the density of regulatory sites in promoters as a measure of the potential and complexity of gene regulation, we found it to be quantitatively the same in all three environments, despite their different physico-chemical conditions and species composition. However, we found that each environment distributes their regulatory potential differently across their functional space. Among the functions with highest regulatory potential in each niche, we found significant enrichment of processes related to sensing and buffering external variable factors specific to each environment, like for example, the availability of co-factors in deep sea, of oligosaccharides in soil and the regulation of pH in the acid mine.

**Conclusions:**

These results highlight the potential impact of gene regulation in the adaptation of bacteria to the different habitats through the distribution of their regulatory potential among specific functions, and point to critical environmental factors that challenge the growth of any microbial community.

**Electronic supplementary material:**

The online version of this article (doi:10.1186/1471-2164-15-877) contains supplementary material, which is available to authorized users.

## Background

Metagenomic studies generate a massive amount of sequence information of communities of organisms living in different physicochemical conditions. This allows, for the first time, to search for the molecular and genetic basis of adaptation through the comparison and the study of genomes of different species sharing the same environment, and of similar species living in different conditions. The comparative studies of the potential protein content in many of these datasets have already provided interesting examples of specific functions that correlate with specific characteristics of the environment. For example, in the search of functional fingerprints related to specific habitats, a comparative analysis between soil, and deep and superficial aquatic environments found abundant orthologous groups specific of these particular habitats [1]. In this case, the examination of higher order processes reveals differences in energy production between these three niches, such as starch and sucrose metabolism in soil or photosynthesis in oligotrophic surface waters [1, 2].

More recently, metagenomic studies have gone beyond the sequencing of DNA and the counting of genes, and have incorporated techniques and protocols to detect, measure and analyze their transcriptome. While the sequencing of metagenomes provides an overview of the genes present in specific environments that can potentially play a role in adaptation, the analysis of expression provides a more precise picture of what functions are expressed and active in a particular moment of the environment. Even though the techniques for mRNA isolation and sequencing from metagenomic samples are still not able to provide comprehensive pictures of expression profiles, there have been important progresses in this direction and some interesting findings. For example, one of the first studies of metatranscriptome, despite it covered a small fraction of the expressed genes, identified specific biological processes active in bacterioplankton communities that could be correlated with either marine or freshwater conditions [3]. As the coverage and accuracy of these analysis increased (mostly by including next generation sequencing techniques), more active processes have been linked to variable environmental conditions. For instance, an expression time-series performed on microbial communities living in surface oceanic showed that processes of energy production were active in hours with light, while anabolic housekeeping processes were predominant during the night [4]. Despite the underlying methodology behind, metatranscriptomics still needs to overcome several challenges [5]. But the rapid progress in this field is promising and we will soon have the opportunity of building accurate expression profiles and compare them across environments, as well as exploring the interaction of processes of different organisms within specific environments.

In the present study we have conducted a novel approach that complements and bridges metagenomic and metranscriptomic concepts. The rationale behind this study relies on the hypothesis that the regulation of the expression of those biological functions that confer adaptation to variable environmental conditions will show higher complexity, i.e. they will have complex regulatory regions.

Previous studies [6, 7] have shown that genes with complex regulation requirements show higher number of transcription factor binding sites (TFBSs) in their upstream cis-regulatory regions compared to housekeeping genes. For example, stress-response genes in yeast need a precise regulation of their expressions patterns to adapt to drastic changes of environmental conditions and also show a significantly higher number of different TFBSs in their upstream regulatory regions. Beyond the extensive analysis of the regulatory characteristics of particular functions [8], up to now, there are not global approaches and studies on how the regulatory potential of entire microbial communities is influenced and organized in natural habitats.

In particular, and using the same rationale, we have measured and compared the complexity of gene regulation in bacteria and archaea living in environments with distinct underlying physico-chemical conditions. For that purpose, we searched within each of the environments for specific functional signatures predicted to have high regulatory potential. These are correlated with specific and also dynamic physico-chemical stress factors of each of the niches. The functional significance of the differences detected highlights the existence of adaptation strategies that rely on the regulatory potential of regions that control the expression of specific fitness genes.

## Results and discussion

With the ultimate goal of identifying and characterizing the extend, to which environmental factors influence the organization of the regulatory potential of particular microbial communities, we have studied and compared the regulome of three fundamentally different ecological niches using whole metagenomic data. We next provide details on the major results and findings of this study: (1) The development of a new pipeline for the identification and prediction of proximal regulatory regions and their TFBS from metagenomic data; (2) and the generation of a collection of regulatory regions from three well studied and reference metagenomic samples (Whale Fall, Waseca Farm and Acid Mine). The comparative analysis of this data has shown that, while (3) the overall distribution of TFBS on promoters is the same across environments, their distribution across their functional space is significantly different, as (4) promoters with higher number of TFBS tend to regulate environment specific functions, and (5) a fraction of these are environment specific and can be linked to characteristic external physicochemical factors (Additional file 1: Figure S1).

### Identification and classification of proximal regulatory regions from metagenomic data

**Figure 1:**
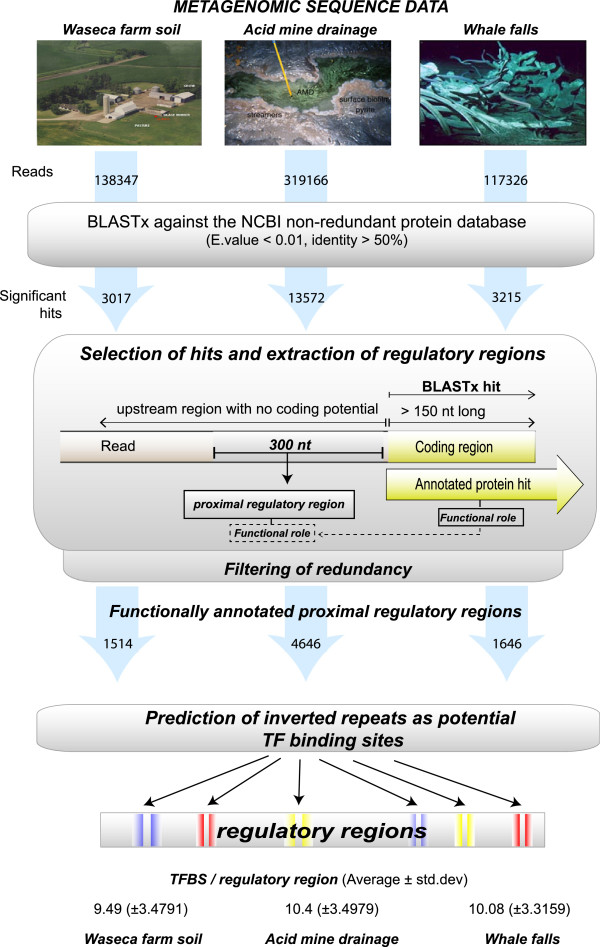
**Computational pipeline generated for the identification and characterization of proximal regulatory regions from metagenomic data.** The figure summarizes the methods used and results obtained in each step of the study.

We first characterized and analyzed the gene regulatory space from metagenomic data obtained from three well-known sequenced environments with clearly different physico-chemical properties: Whale Fall Community, Acid Mine and Waseca County Farm Soil [1]. For that, we started by identifying and defining gene regulatory regions to later characterize them, as to their levels of TF binding, i.e. their regulatory potential. For the design of a search strategy, we followed two major considerations: first (1) avoiding biases in favor of most abundant and well-known bacteria (and closely related species), as well as, (2) ensuring an equal coverage through all the phyla detectable in those samples. As a result, we developed a pipeline that consists of two major steps: (1) first the identification of proximal regulatory regions and then, within each of them, (2) the prediction of potential regulatory transcription factor binding sites. The complete pipeline is detailed in the Methods and summarized graphically in Figure 1.

Through extensive homology searches, our procedure identified putative proximal regulatory regions in Waseca Farm Soil, in Acid Mine Drainage, and in the Whale Falls Sample (a complete catalogue of these regions can be found in Additional files 2, 3 and 4). A first and basic taxonomical analysis of these sequences shows that these promoters cover all phyla (Additional file 1: Figure S2) that were previously described in these environments [1].

Next, we estimated the level of regulatory potential for each of these promoter regions through the prediction of their transcription factor binding sites. In order to minimize possible biases favoring promoters from well-studied bacteria (or close species), we did not consider TFBSs prediction strategies that rely on the homology mapping of described TFBSs. Instead, we used a *de novo* prediction protocol that relies on the identification of palindromic repeats [9], which have been previously determined as preferred binding sites for transcription regulators in bacteria and archaea [9–14]. Because this method was originally developed for the analysis of single genomes [9] and, although it has been applied to a wide variety of bacterial sequences and studies [14–16], we needed to adapt it to cope with the heterogeneity and redundancy of metagenomic data by including some modification in the scoring system.

### Evaluation of predicted promoters and TFBSs

Like any other *de novo* prediction method in sequence analysis, we have to initially assume the presence of false positive TFBS models among correct predictions. To assess for the reliability of all of our predictions and to put our strategy and results into the context of our goals and of the current knowledge about regulatory regions in prokaryotes, we performed different quantitative and qualitative comparisons with available independent data and methodologies.

From a quantitative point of view, we (1) first observed that the global average of 10 TFBS per promoter (with 0 as minimum and 25 as maximum values) that we identify from all three environments is in agreement with previous estimates obtained with different bacterial species and methodologies. For example, using genome comparative analysis, an average of 11–13 TFBS motifs per promoter was found for *Shewanella*[17]. In addition, a study of the transcription regulatory network of *E. Coli K12* predicted up to 16 sites per promoter [18], and up to 20 through the identification of half-sites motifs [19]. (2) We also evaluated the performance of our methodology by comparing our results with those obtained with an independent method, MotifClick, that predicts cis-regulatory regions using a graph-based polynomial-time algorithm [20]. After running both predictors over intergenic *E. Coli* regions, we observed that the densities of TFBS resulting from one or the other strategy showed high correlation values (rho = 0.52, p-value < 2.2 × 10^−6^; (Additional file 1: Figure S3).

**Figure 2:**
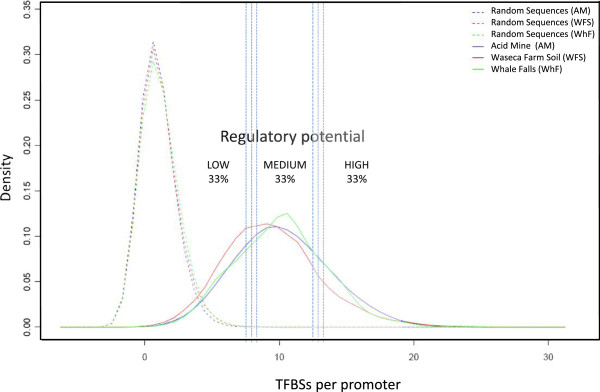
**Distribution of regulatory regions predicted per each environments analyzed in this study according to their content of TFBSs.** Solid lines represent the distributions of TFBS obtained using real data, whereas dashed lines show predictions using randomized DNA sequences, i.e. with no biological information. Three major groups of regulatory potential are also shown: low, medium and high, which corresponds to the three bins analyzed in Additional file 1: Figure S4.

From a qualitative point of view, we first (1) assessed the biological significance of our predictions by carrying out a randomization test consisting in applying the same prediction pipeline to our collection of promoters with their nucleotide sequence completely shuffled, i.e. with no biological information. We observed that the distributions of the number of motifs per promoter were significantly different between the real and the randomized sample (Figure 2). (2) Furthermore, we screened for coincidences between our predicted TFBSs and those reported in the RegPrecise database [21], which consist on manually curated site reconstructions in various bacteria genomes. This comparison showed that 28% of our predicted binding sites include, at least, one possible binding sequence of the matrices for each of the 38 TFs included in RegPrecise (Additional file 5). (3) Finally, we also searched for a particular type of false predictions, which consist on regulatory palindromic repeats with no binding potential, named Clustered Regularly Interspaced Short Palindromic Repeats (CRISPRs) [22]. The results that we obtained using the CRISPRFinder web tool [23] showed a negligible amount of these regions (less than 1% of our set of promoters), which were subsequently removed from the analysis.

In summary, all these evaluation tests suggest that our set of promoters is both, quantitatively and qualitatively reliable, as they show a significant fraction of reported TFBS, and a small portion of false positives. But, most importantly, the presence of this small fraction of false positives is not expected to affect our final conclusions, as these come from comparisons within and between environments and do not rely on absolute TFBSs counts.

### Functional organization of regulatory potentials within each environment

We then studied how microbial communities living in these environments organize and distribute their regulatory potential through the different biological functions and to which extend this could obey to specific adaptation needs. It is interesting to observe that, whereas the range of density of predicted sites per promoter is wide within each of the environments, the overall distribution and the averages are similar: 9.98 (±3.29), 9.58 (±3.49) and 10.28 (±3.35) for Acid Mine Drainage, Waseca Farm Soil and Whale Falls samples, respectively (Figure 2). This indicates that, although these three environments present (1) different sequence coverage, (2) different physicochemical characteristics and (3) different species composition, the overall regulatory potential, as to the total number of different TFBS, and their distribution across the promoters follow a similar pattern.

To go beyond simple counts and to explore whether or not this regulatory potential is distributed equally through all the functions of each of the metagenomes, we first identified the functions under the control of our collection of proximal regulatory regions. For this, we assigned to each promoter the functional category (from SEED database) [24] of the corresponding downstream coding region using MEGAN [25] see (Additional files 6, 7 and 8, for a complete list of functions and TFBS densities). We first investigated whether the regulatory potential is organized differently over the functional space of each of the environments. For this, we ranked all promoters of each sample according to their TFBS density and count, for each density group, how many associated functions are specific of that particular environment, or co-occur in one or in the other two samples. This analysis showed significant differences between promoters. Interestingly, the functions under the control of promoters with high number of TFBSs show significantly less co-occurrences between environments, than those regulated by promoters with lower regulatory potential. The fact that promoters with high density of TFBS are enriched in environment specific functions provides the first hint that processes that require complex regulation might provide adaptation to environment specific variable external factors. (Additional file 1: Figure S4). We expect that a large fraction of functions that showed a higher co-occurrence among environments likely correspond to housekeeping roles.

To study this further, we next investigated which functions are specifically enriched among the highly regulated ones in each of the environments. For this, we zoomed into the fraction of the 33% highest regulated promoters (i.e. with more than 12 TFBSs/promoter) and subdivided it further into subgroups covering the 1, 5, 10, 20, 30 and 40 top percentages of TFBS density, to finally analyze the functional enrichments within each of them. This analysis highlighted different enriched functions in each of the environments (see Additional file 1: Figure S5 (Acid Mine), S6 (Waseca Soil), S7 (Whale Falls)). These enriched functions cover different types of processes, the majority of them involved in sensing and buffering external factors, such as, receptors and transporters in Acid Mine and stress response systems in Whale Falls.

### Potential environment-gene regulation relationships

In order to finally highlight potential points of interaction between highly regulated functions that could provide adaptation to variable conditions specific to each of the environments, we first selected for each habitat, those functions that show stronger enrichment, i.e. with pvalue < 0.05, among the top 1, 5, 10 and 20% groups and with clear orthologous functions in the other two samples. This subgroup of functions include (virulence, cell cycle, carbohydrates metabolism, stress response and cofactors metabolism), which we then compared among environments and evaluated their relationship with the niche specific variable factors. For this, we carried out extensive literature searches on different biochemical mechanisms of adaptation guided by these functions and the characteristics of the environment. Despite the limited information about the environment physico-chemical factors characteristic of available metagenomic studies, we propose in the following sections potential adaptive scenarios by correlating highly regulated functions with known variable external factors in each of the environments.

### Waseca Farm Soil

In Waseca Farm Soil, carbohydrates metabolism related functions appear as highly regulated, more precisely di and oligosaccharides metabolism (pvalue = 1×10^−16^, within environment and adjusted pvalue (Bonferroni) = 9 × 10^−13^ for Fisher’s exact test between groups). This fact could be in concordance with the fluctuations in organic matter concentrations in the soil, such as, plant debris, which has also been previously proposed as an explanation for the presence of other carbohydrate metabolism functions specific of this environment [1]. This further agrees with the behavior observed in lower eukaryotes abundant in soil, like yeast, where high complexity in their transcriptional regulation were found upstream of genes that play a role in carbohydrates metabolism [26]; and with the fact that, in this niche, the upstream region of the FruR gene, a known TF that regulates carbohydrate metabolism, appears as highly regulated, with the highest number of predicted TFBS (Additional file 1: Figure S8).

### Whale Falls samples

**Figure 3:**
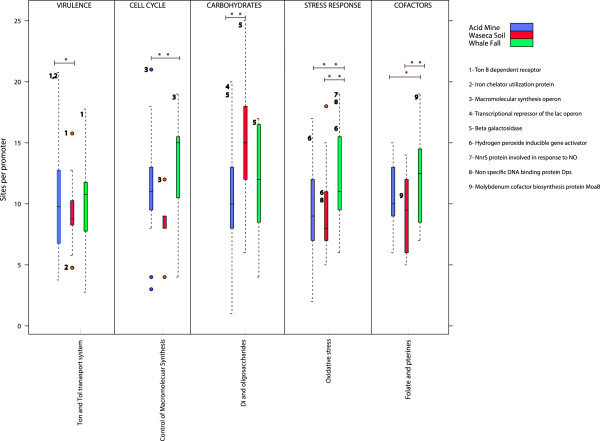
**Box-plots showing the TFBSs density per promoter related to highly regulated process in Acid Mine, Waseca Soil and Whale Falls samples.** The * indicates pvalue ≤ 0.05, and the ** indicates pvalue ≤ 0.01 (Fisher’s exact test) between the groups where the line is drawn. The numbers and functions listed at the right side represent the genes with highest regulatory potential. Their respective positions in the box-plot obey to their average level of regulation in a particular environment. In the X axis, are written the parental (above) and specific enriched group of functions (below) according to SEED classification.

A different scenario is observed in Whale Fall where, even though each of the subsamples were collected in a specific moment of decomposition from two different whales and at different depths, they all share similar general physico-chemical patterns, predominating the drastic fluctuations of nutrient availability [1]. In agreement of what would be expected for microorganisms living in these kind of environments, most of the highly regulated functions that are enriched in whale falls samples are related to adaptation capabilities to starving periods (Figure 3). Particularly, we found TFBS rich promoters upstream of genes that are involved in cell cycle and growth, i.e. the control of basic macromolecular synthesis operon. This is in contrast to what happens in Waseca and Acid Mine, where the same functions present lower density of TFBSs (Figure 3).

Moreover, bacterial communities living in cold water are also exposed to high concentrations of oxidant reagents [27] causing an increase in the metabolic costs associated with the activation of antioxidant defenses. In fact, functions related with the response to oxidative stress appear specifically enriched in this environment compared to others. These functions comprise, for example, the hydrogen peroxide-inducible gene activator and a hem and copper containing membrane protein (NnrS), that needs to respond to external NO concentrations. Additionally, parts involved in the machinery that protects genomic DNA during prolonged non-growing phases [28], like the non-specific DNA binding protein (Dps), also appear as highly regulated in this niche.

It has been also pointed before, that the uptake and metabolisms of cofactors and amino acids are particularly variable in marine environments, essential to adapt to typical oceanic oligotrophic conditions [2]. In agreement with this, cofactor metabolism related functions are also enriched (adjusted pvalue (between groups) ≤ 0.05). In particular, we found enrichment for enzymes involved in the metabolism of molybdenum cofactors, pterin and folate (Figure 3). These findings were further confirmed by the overrepresentation of TyrR and ArgR binding sites in this niche, both known to be TFs involved in the control of amino acid transport for the synthesis of proteins (according to the RegPrecise database; see Additional file 1: Figure S8).

### Acid Mine

**Figure 4:**
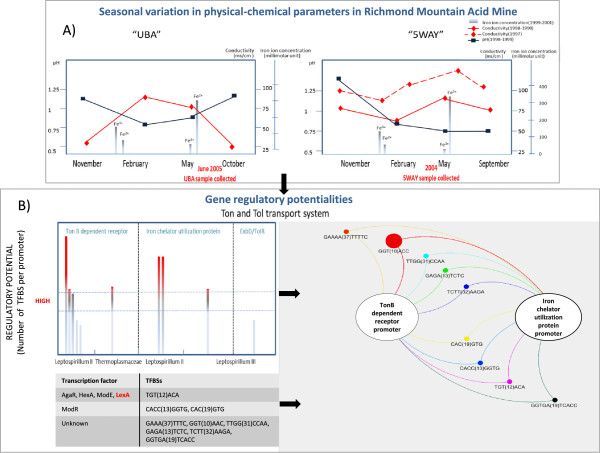
**Example of the relationship between environmental factors and organization of regulatory potential in Acid Mine. A)** Seasonal variation in pH, conductivity and iron concentration across the year. The left panel represents samples from “UBA” and right panel represents the samples collected from “5WAY” (data taken from [29]). **B)** TFBSs found in the promoters which regulate Ton and Tol transport system genes. The left panel shows a graphic illustrating the regulatory potential related to Ton and Tol transport system in Acid Mine (“UBA”). The promoters are grouped by function and species (X axis). Each bar in the graphic represents a promoter and, the red color in the bars indicates that the number of sites per promoter is equal or more than fifteen. Below are represented the most abundant palindromic sites found in those promoters, some of them corresponding to known TF, such as, LexA, ModR, AgaR, etc. The right panel represents a network of TFBSs shared between promoters of TonB dependent receptor and iron chelator utilization protein; both proteins are represented by white circles. The TFBSs are represented by colored circles with size proportional to the number of sites found.

The acid mine is characterized by extreme physico-chemical conditions, showing low pH records and fluctuating temperature, conductivity and rainfall (see Figure 4A) [29]. Among the functions with high regulatory potential that appear enriched in this niche are those known to play a role in the adaptation to changes in external osmolarity, typical of environments with variable distribution of rainfall across the year [30, 31] (Figure 4A). It is worth mentioning the high regulatory potential of some genes related to the TonB transport system (Figures 3, 4B), which are also involved in avoiding toxicity by keeping metal homeostasis inside the cell [32], in particular of iron. The high regulatory potential of the TonB-dependent receptor and the iron chelator utilization protein (Figure 3) might provide homeostasis (i.e. plasticity) to acid mine bacteria living under variable ferric concentrations, which is further confirmed by the fact that a significant fraction of homeostasis-related promoters could be assigned to *Leptospirillum* (genus known to be adapted to low pH [33]) (Figure 4). In addition, we found overrepresentation of binding sites for LexA transcription factor in this niche (see Additional file 1: Figure S8), and, specifically in Ton and Tol transport systems related promoters (the sequence for LexA binding site is in Figure 4B, colored in red). LexA transcription factor is known to be involved in the response to DNA damage and external pH fluctuations [34]. In fact, when we evaluated the fraction of binding sites shared between two members of the Ton and Tol system (iron chelator utilization protein and TonB dependent receptor), we found a high number of coincidences for other sites besides LexA (i.e. sites for the transcription factors ModR and ModE involved in metal metabolism) (Figure 4B).

Taken together, the fact that highly regulated functions are not the same between the different environments agrees with previous metatranscriptome studies [3, 4] and indicates that the organization of the regulatory potential between the functional space of each niche is different and influenced by the environmental physico-chemical conditions. This could reflect organism-environment interaction points where gene regulation should be able to provide enough plasticity to the functional network for the adaptation to variable external parameters.

## Conclusions

We have here studied how variable physico-chemical conditions of the environment can shape the regulome of microbial communities living there to provide adaptation. We have combined existing and novel methodologies and applied it to three environments (Acid Mine, Whale Fall and Waseca Farm Soil) to identify and characterize, for the first time, their regulatory space, i.e. proximal promoters and their corresponding TFBSs. Taking the density of TFBSs as a measure of the level of regulatory potential, we first observed that, despite the differences of the living conditions of each of the environments studied here, their distribution of the regulatory potential, at quantitative level, appears to be nearly identical. However, when we went beyond simple counts we observed that the associated cellular functions in different groups related to the regulatory potential tend to be environment specific. This supports our hypothesis and expectation that point to a role of gene regulation in the adaptation of organisms to particular and variable external factors. Also in this direction, we have found specific functional enrichments among highly regulated functions in each of the metagenomas, suggesting potentials interaction points between gene regulation and dynamic environmental conditions. In particular, we have identified points of interaction between signatures of significant functional enrichment and specific characteristics of the marine and terrestrial environments. These results highlight the impact of gene regulation in the adaptation of microbes to their habitat. Beyond contributing to the general understanding of how wild bacterial communities interact with the environment, our methodology can also be used to identify potential external factors to which bacteria are particularly sensitive in order to design efficient communities for therapeutic, or ecological needs.

## Methods

### Datasets

Metagenomic samples (i.e. Sanger sequencing reads) were downloaded from the Camera Database [35]. In particular, (1) samples of whale falls were obtained from three independent libraries named Whale falls: CAM_SMPL_WHALEFALLBONE (Whale fall carcass bone, W. Antarctic Peninsula Shelf), CAM_SMPL_WHALEFALLMAT (Whale fall carcass microbial mat, Santa Cruz Basin), and CAM_SMPL_WHALEFALLRIB (Whale fall carcass rib bone, Santa Cruz Basin) in the database. These three sets differ in the depth of the sampling and come from two different whale samples. (2) The Acid Mine dataset is formed by 5-Way (CG) Acid Mine Drainage Biofilm Metagenome and UBA Acid Mine Drainage Biofilm Metagenome reads. The first corresponds to a low-complexity microbial biofilm growing hundreds of feet underground within a pyrite (FeS_2_) ore body. The UBA biofilm was subaerial, collected from the base of a ~2 m high pile of pyrite sediment. (3) The third environment corresponds to a surface soil (0–10 cm) collected from a Waseca County farm in Minnesota.

### Promoter identification

The prediction and classification of regulatory regions from metagenomic data relies on the extraction of DNA regions upstream of coding genes detected through homology searches directly from the sequencing reads. For this protocol we selected conservative filters to ensure the reliability of the putative promoters found. Simplified in Figure 1, our protocol consisted in: (1) filtering out reads shorter than 800 base pairs. This filter keeps up to 90% of all reads and ensures both, the detection of the coding region and the extraction of the putative promoter from the reads; (2) detection of reads with coding potential through the comparison of all the sequences of each metagenome with all bacterial and archaeal annotated proteins (NCBI; http://www.ncbi.nlm.nih.gov/Ftp/), using BLASTx (default parameters [36], and selecting those reads with a match to a known protein over, at least, 150 amino acids and with more than 50% of sequence identity; (3) filtering out those positive reads that did not contain at least 300 nucleotide of non-coding sequence upstream of the region matching in BLASTx. This filter enriches our sampling in regions with regulatory potential by avoiding internal genes of operons, which are expected to have short upstream regions with no regulatory potential. Finally, from the remaining accepted reads (13572, 3017, and 3215, for Acid Mine Drainage, Waseca Farm Soil and Whale Falls Samples, respectively) we extracted 300 nucleotides upstream of the coding region as putative promoter sequence. We expect that the 300 base pairs criteria will affect equally all bacteria and environments and will not favor bacteria with largest genomes, as this length has been also described for *Pelagibacter ubique*, the free living bacteria with the smallest genome known [37]. Moreover, fixing this length also avoids short intergenic regions within operons, as their regulatory role is not yet well understood.

To avoid other possible biases favoring common species in these environments and to make possible comparative and qualitative analyses between them, we also removed the redundancy within these collections of putative promoter sequences using a cutoff of 98% of sequence identity. We also removed those reads that correspond to eukaryotic DNA, mostly from plant species in the Waseca sample, identified using MEGAN [25]. To discard the inclusion of (parts of) ncRNA genes into the collection of promoters, we applied a second filter to remove ncRNAs that target untranslated 5’ portions of mRNAs by using Rfam [38] and also we did a second prediction of coding region in our set of putative promoters using the software Prodigal [39] that allows the identification of genes even if the specie is unknown.

### Prediction of transcription factor binding sites

We next searched for sequence motifs with binding potential within the putative promoters identified before. For this, we used a *de novo* prediction method that is based on the identification of palindromic repeats separated by a spacer DNA region. In particular, we used the most recent adaptations of the method [14] originally described by Li and coworkers [9].

In order to identify putative cis regulatory elements, we screened each promoter sequence for *W*_1_*NW*_2_, DNA motifs, where *W*_*1*_ and *W*_2_ are 3–5 nucleotide long palindromic sequences separated by *N* (0–30) arbitrary bases. This method relies on the fact that prokaryotic TFBSs are usually palindromes between 12 and 30 base pairs, which may facilitate the dimerization and binding of TFs [12].

To assign a probabilistic values to all motifs found, we first calculated the probability of observing *n*(*D*) copies of a dimer *D* by chance, by pooling all the promoters and calculating its expected frequency from the formula,
1

where *n*(*W* 1) and *n*(*W* 2) are the total number of occurrences of *W*_1_ and *W*_2_ in the whole data set (all three environments together) and *L*eff(D) = ∑*r*(*L*(*r*) − *L*(*D*) + 1) is the number of independent positions in the data where a motif *D* of length *L*(*D*) can be found. The summation is over all the occurrences among 11,614 promoters identified, each with a length *L*(*r*) (i.e. the estimated distance between coding regions). Finally, a *P*-value is assigned to each of the motifs assuming that the background follows a Poisson distribution:
2

and is considered significant if *P* < 1/*N*_*motif*_, where *N*_*motif*_ is the total number of positive motifs found. As *W*_1_ is the reverse complement of W_2_ (palindrome), the cutoff on *P* is corrected by the total number of palindromic dimers found [9, 14].

In order to identify environment specific enrichment of our know TFBSs (i.e. those present in the RegPrecise database), we run a Kruskal Wallis test to compare the density of each particular known TFBS among all three environments. The density of known TFBS per metagenomes is calculated as follows:
3

where D (x) is the density of TFBSs per metagenome, N represents the number of promoters found in the x metagenome and Tbp is the number of base pairs per promoter (300 base pairs). The complete list of overrepresented TFBSs found in our selected promoter set are shown in Additional files 6, 7, 8, 9, 10 and 11, for Acid Mine, Waseca Farm Soil and Whale Falls samples, respectively.

### Method validation

For the randomization test on TFBS prediction, we run the corresponding searching methodology on predicted promoter regions after shuffling their sequence using a 20 nt window to ensure the minimum variance of local nucleotide composition.

For the comparison with the MotifClick method [20] we first downloaded intergenic regions from the *Escherichia coli K12-W3110* genome from IMG database (https://img.jgi.doe.gov). We ran MotifClick (motif length = 14 nt) over these regions, specifically 300 nucleotide upstream annotated TSS and recorded the number of positive predictions per promoter. These values were then compared with the results provided by our method applied on the same set of *E. Coli* regulatory regions (Additional file 1).

### Statistical procedure for the functional analysis

Functional assignment for all the data was performed by MEGAN software [25] using the output of BLAST searches of our reads against databases of known bacterial proteins. Through this comparison we could identify up to 1646 (Whale Falls Samples), 4646 (Acid Mine Drainage) and 1514 (Minnesota Farm Soil) gene upstream segments with functional assignment. In order to roughly study up to which level low, medium and high regulated functions are shared among environment we have run a Spearman test for independence using R, for the rectangular plot and correspondence analysis we use the plot function included in R graphics (http://www.r-project.org/) (see Additional file 1: Figure S4).

In addition, functional enrichment analysis was done by first ranking all promoters as to their number of predicted TFBSs. Then, for each of the groups of interest, we ran a Fisher’s exact test for count data to see whether particular functions within each group (top 1%, 5%, 10%, 20%) were specifically enriched versus the total distribution of functions. For this, we have used “all intermediate” functional levels according to MEGAN classification. Heat maps for all function within environment were obtained using package ggplot2 for R (Additional file 1). Then, we retained significant cases based on two criteria 1) functions whose p < < 0.05 within environment and 2) functions with orthologous in the other three environments. Those selected groups were compared again, this time among environments, for this analysis we ran a Fisher’s exact test to see whether functional enrichment within environment were maintained among them.

## Electronic supplementary material

Additional file 1: Figure S1: Shows the overview of the general results of this study. **Figure S2.** shows the comparative analysis of the taxa obtained with MEGAN on our promoter regions compared with that obtained previously using 16S rRNA information from the same samples in Waseca soil (a), Whale falls (b), and Acid mine (c). **Figure S3.** represents the correlation analysis between the TFBSs predictions per promoter using the method explained in this paper versus MotifClick predictions. **Figure S4.** illustrates a global view of the relationship between regulatory potential and the level of co-occurring functions within each of the environments. **Figure S5.** Results of the functional enrichment analysis for Acid Mine using the predefined bins. **Figure S6.** Results of the functional enrichment analysis for Waseca Farm using predefined bins. **Figure S7.** Results of the functional enrichment analysis for Whale Falls using predefined bins. **Figure S8.** shows the relative abundances of our TFBS prediction that matched known TFBS. (PDF 4 MB)

Additional file 2: **List of promoters selected after applying the methodology described in Figure** 1 **on Waseca Farm Soil data.** (ZIP 261 KB)

Additional file 3: **List of the promoters selected after applying the methodology described in Figure** 1 **on Acid Mine data.** (ZIP 817 KB)

Additional file 4: **List of the promoters selected after applying the methodology described in Figure** 1 **on Whale Fall Samples data.** (ZIP 264 KB)

Additional file 5: **Table listing the number of TFBSs per genomes found after applying our method versus the number of sites described in Regprecise database.** (PDF 88 KB)

Additional file 6: **Table in CSV format listing the number of TFBSs identified for each promoter and the function assigned to the corresponding downstream coding region in Acid Mine.** (CSV 542 KB)

Additional file 7: **Table in CSV format listing the number of TFBSs identified for each promoter and the function assigned to the corresponding downstream coding region in Waseca Soil.** (CSV 172 KB)

Additional file 8: **Table in CSV format listing the number of TFBSs identified for each promoter and the function assigned to the corresponding downstream coding region in Whale Falls.** (CSV 151 KB)

Additional file 9: **A list (CSV MS-DOS format) of overrepresented TFBSs per promoter found in Acid Mine, Waseca Soils and Whale falls, respectively.** The abbreviated nomenclature used for the binding sites is the following: N, W, Sequence, where N is the number of variable nucleotides. W is the number of nucleotides defining the inverted repeat. Sequence is the actual sequence of the site. Example: 10 3 ATC, corresponds to: ATCNNNNNNNNNNGAT. (CSV 3 MB)

Additional file 10: **A list (CSV MS-DOS format) of overrepresented TFBSs per promoter found in Acid Mine, Waseca Soils and Whale falls, respectively.** The abbreviated nomenclature used for the binding sites is the following: N, W, Sequence, where N is the number of variable nucleotides. W is the number of nucleotides defining the inverted repeat. Sequence is the actual sequence of the site. Example: 10 3 ATC, corresponds to: ATCNNNNNNNNNNGAT. (CSV 873 KB)

Additional file 11: **A list (CSV MS-DOS format) of overrepresented TFBSs per promoter found in Acid Mine, Waseca Soils and Whale falls, respectively.** The abbreviated nomenclature used for the binding sites is the following: N, W, Sequence, where N is the number of variable nucleotides. W is the number of nucleotides defining the inverted repeat. Sequence is the actual sequence of the site. Example: 10 3 ATC, corresponds to: ATCNNNNNNNNNNGAT. (CSV 1 MB)
