# Supplementary material for: Adaptation to environmental factors shapes the organization of regulatory regions in microbial communities
Source: BMC Genomics. 2014 Oct 8;15(1):877. doi: 10.1186/1471-2164-15-877 (PMC4287501; doi:10.1186/1471-2164-15-877)
Supplement: Supplementary file 1 — Additional file 1: Figure S1: Shows the overview of the general results of this study. Figure S2. shows the comparative analysis of the taxa obtained with MEGAN on our promoter regions compared with that obtained previously using 16S rRNA information from the same samples in Waseca soil (a), Whale falls (b), and Acid mine (c). Figure S3. represents the correlation analysis between the TFBSs predictions per promoter using the method explained in this paper versus MotifClick predictions. Figure S4. illustrates a global view of the relationship between regulatory potential and the level of co-occurring functions within each of the environments. Figure S5. Results of the functional enrichment analysis for Acid Mine using the predefined bins. Figure S6. Results of the functional enrichment analysis for Waseca Farm using predefined bins. Figure S7. Results of the functional enrichment analysis for Whale Falls using predefined bins. Figure S8. shows the relative abundances of our TFBS prediction that matched known TFBS. (PDF 4 MB) [file 12864_2014_6783_MOESM1_ESM.pdf]

Supplementary figure 1

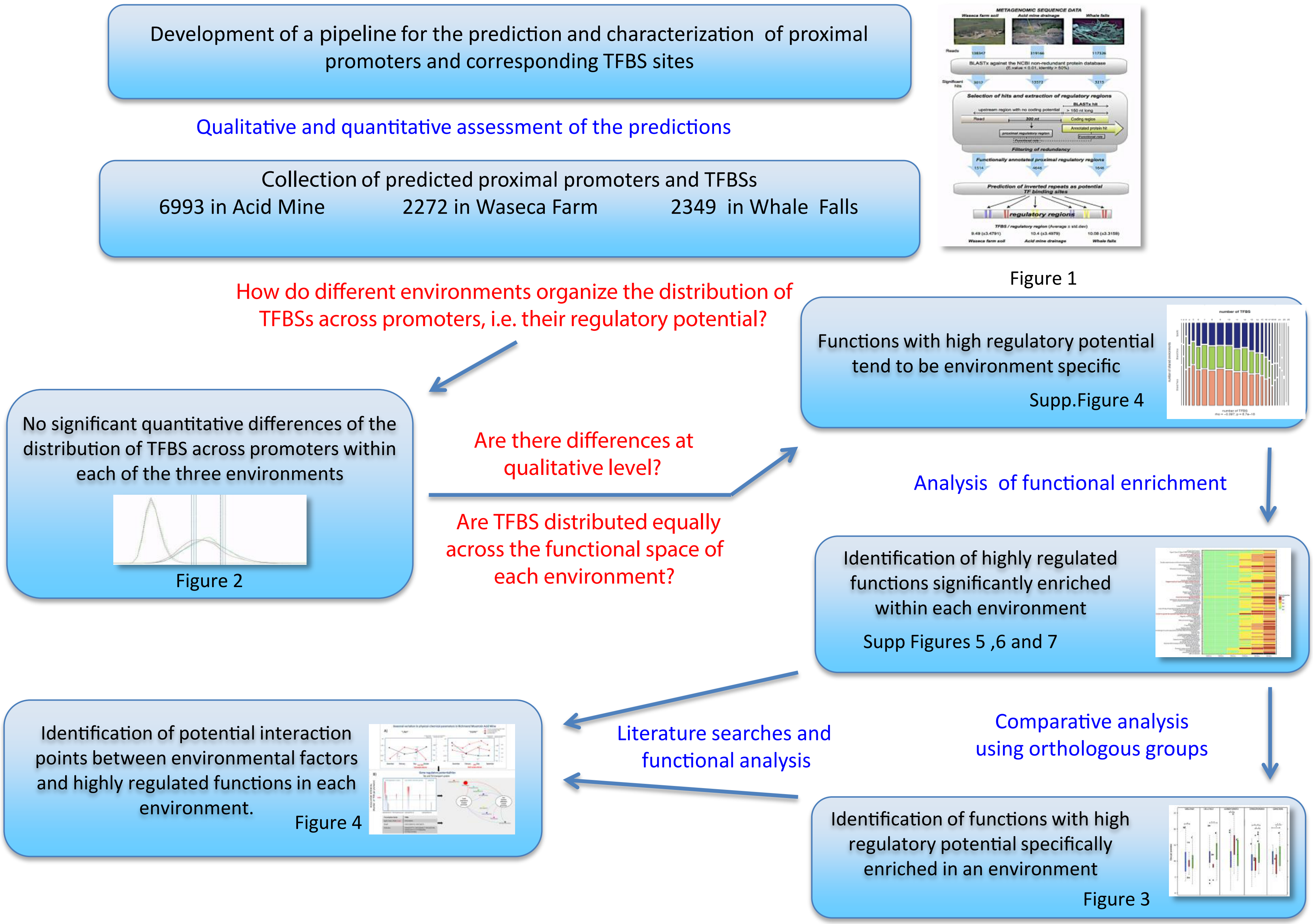

Supplementary figure 1. Overview of the study and major results obtained.

Supplementary figure 2

A)

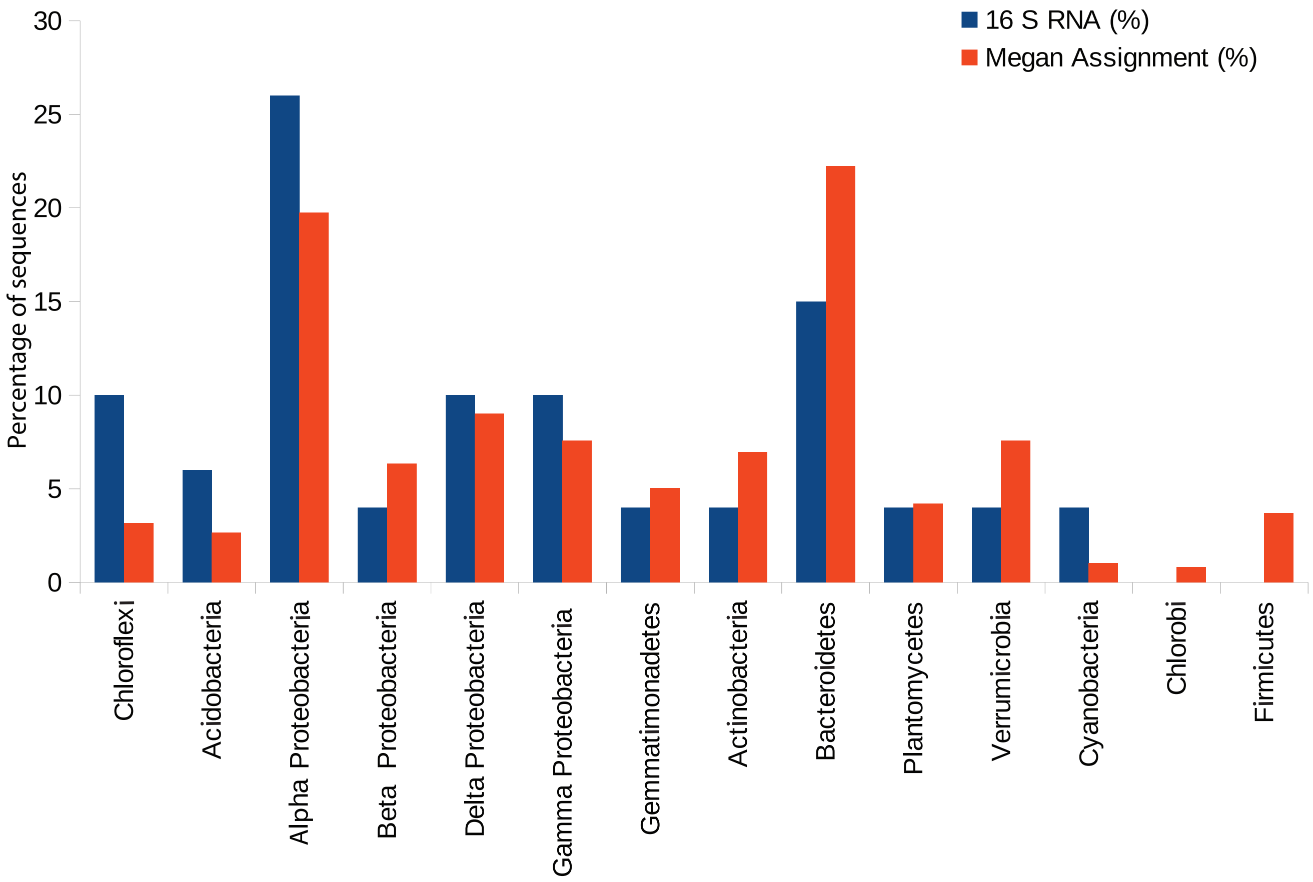

B)

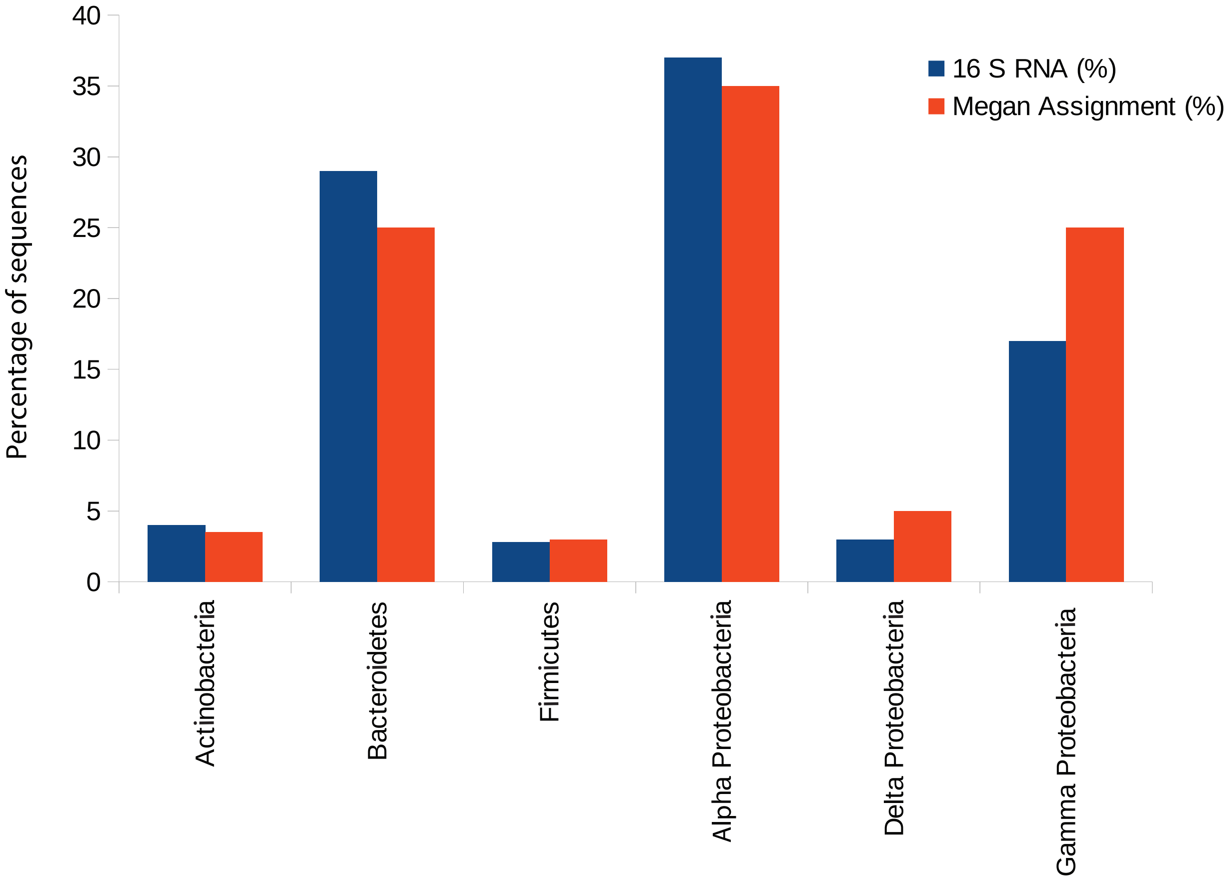

Supplementary figure 2

C)

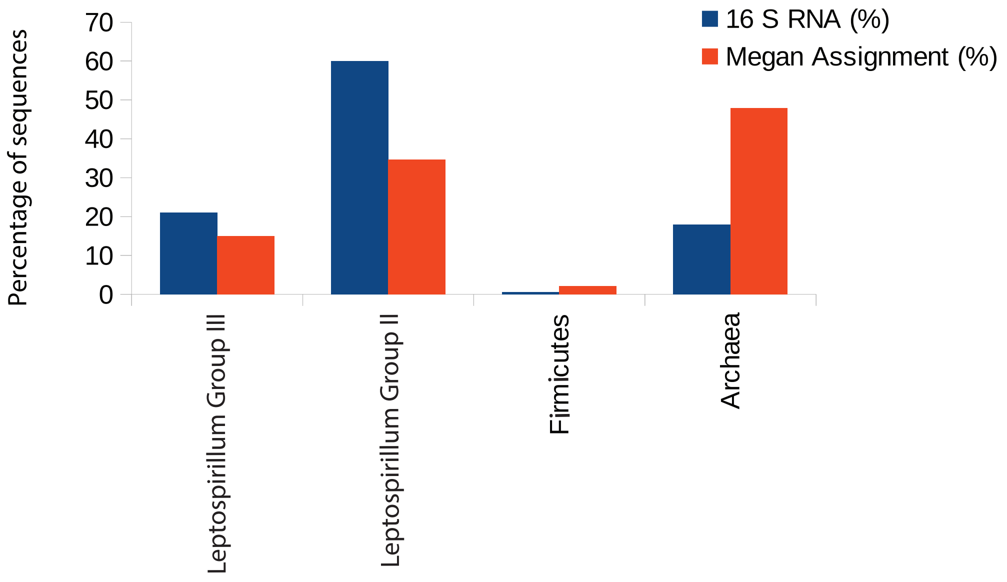

Supplementary figure 2. Analysis of the coverage of taxa of our identified promoters using MEGAN (red columns) in comparison with the taxa previously identified using 16 S rRNA for the same samples (blue columns). (A) Waseca soil (B), Whale falls, and (C) Acid mine. Descriptions of taxa were obtained from Tringle et al., 2005 for Waseca Soil and Whale Fall, and from Tyson et al, 2005 (5 way) and Lo et al, 2007 (UBA) for Acid Mine.

Supplementary figure 3

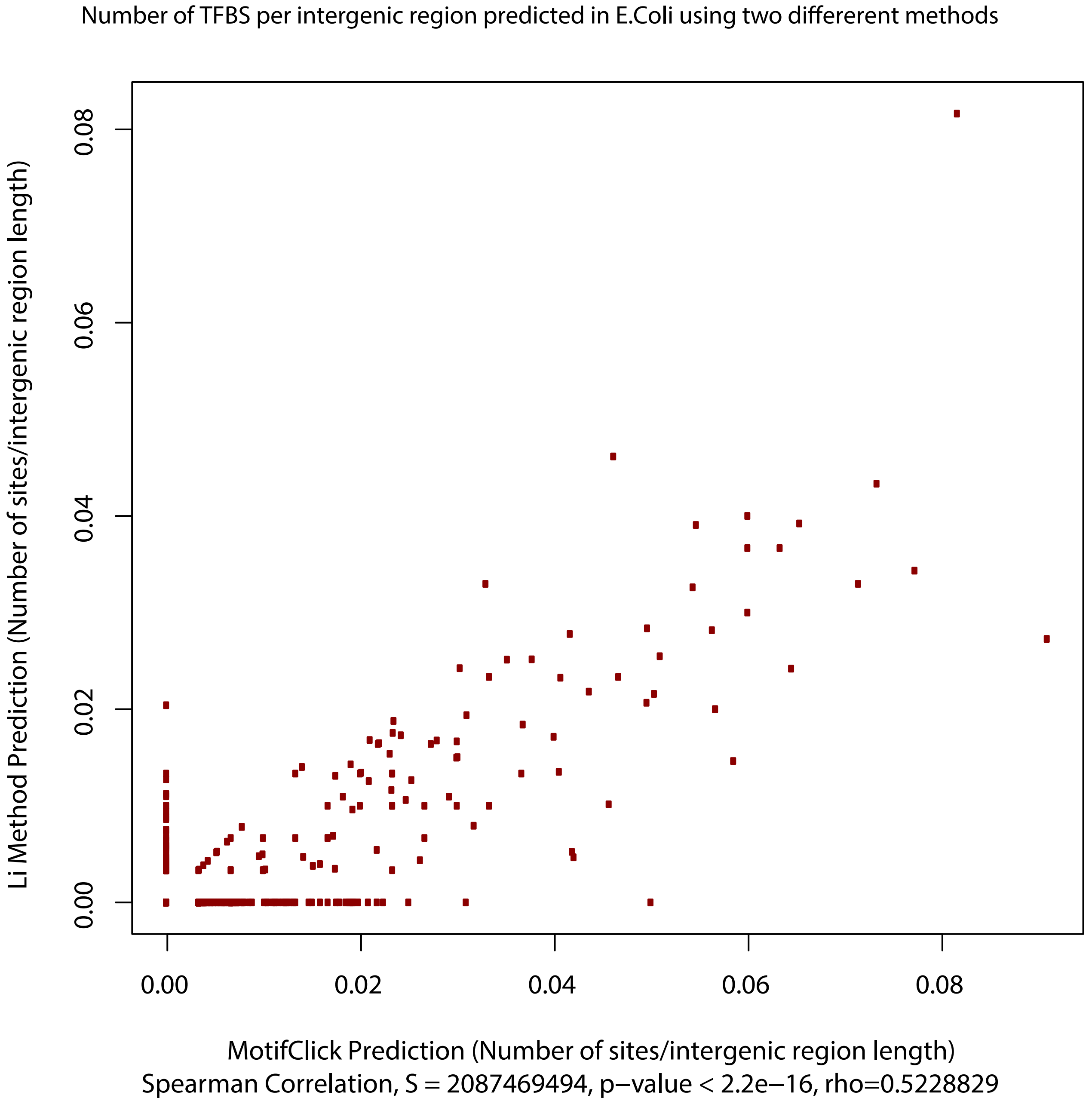

Supplementary figure 3. Correlation analysis between the TFBSs predictions per promoter using the method explained in this paper versus MotifClick predictions. For this comparison, both methods were applied to the same E.coli genome.

Supplementary figure 4

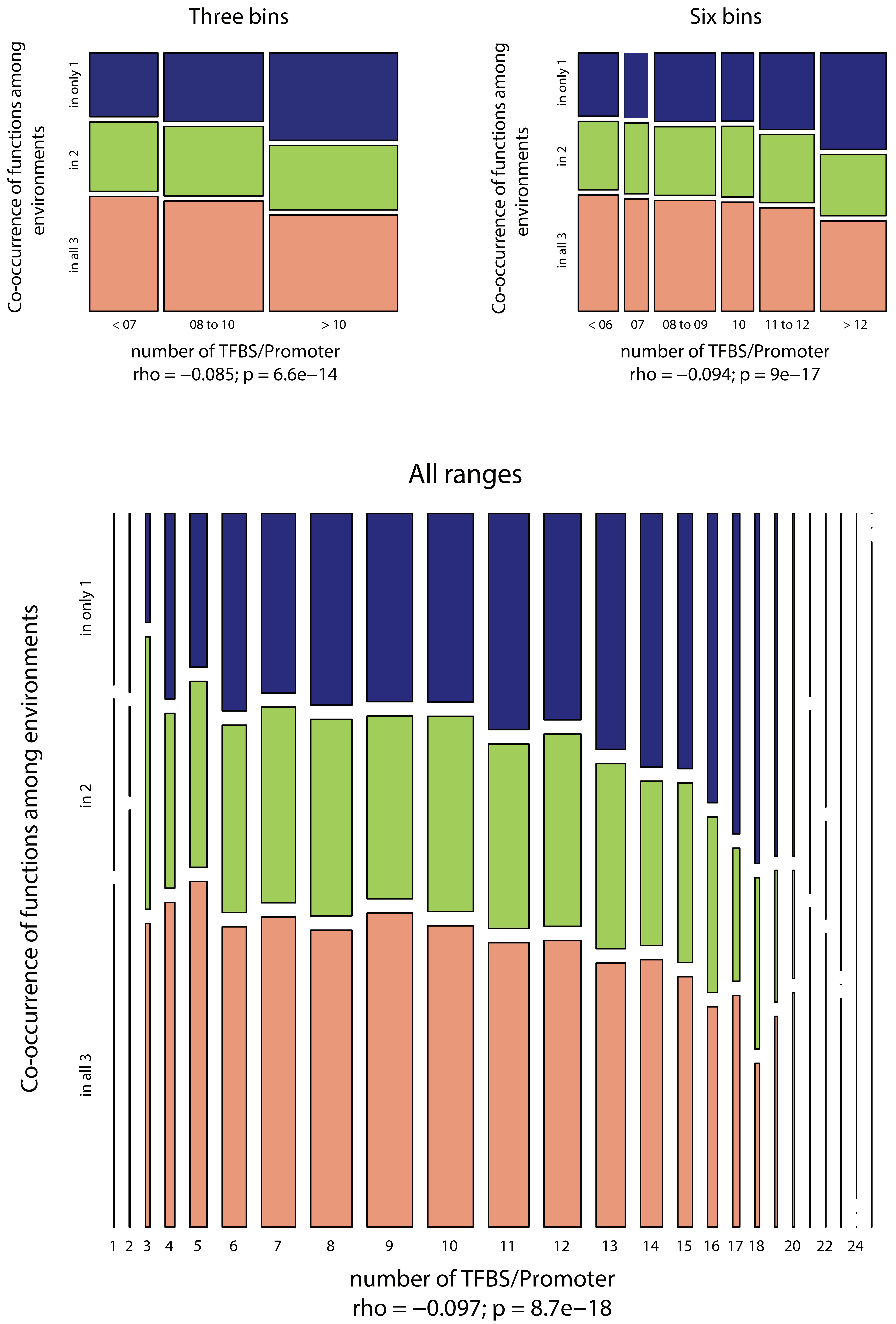

Supplementary figure 4. Spearman correlation analysis at different resolutions or binning size : 3 bin, 6 bin and considering all the ranges of TFBSs densities obtained (0 to 25). The mosaic plots show a global view of the relationship between regulatory potential and the level of concurrency per function within each of the environments.

Supplementary figure 5

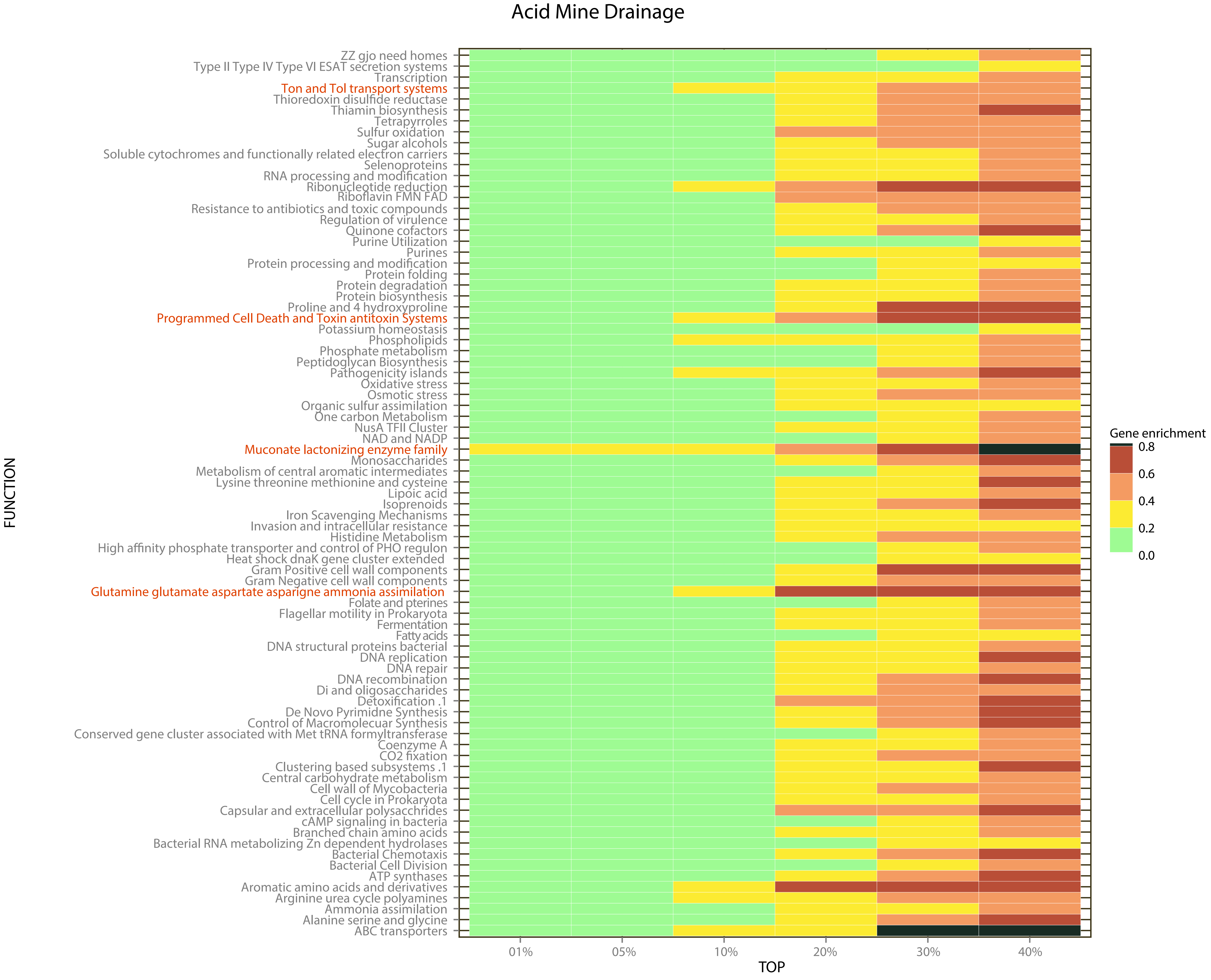

Supplementary figure 5. Results of the functional enrichment analysis for Acid Mine Dranaige using the predefined bins (TOP: 1, 5, 10, 30, 40 %). Functions with a significant enrichment in the top 33% bin are shown in red.

Supplementary figure 6

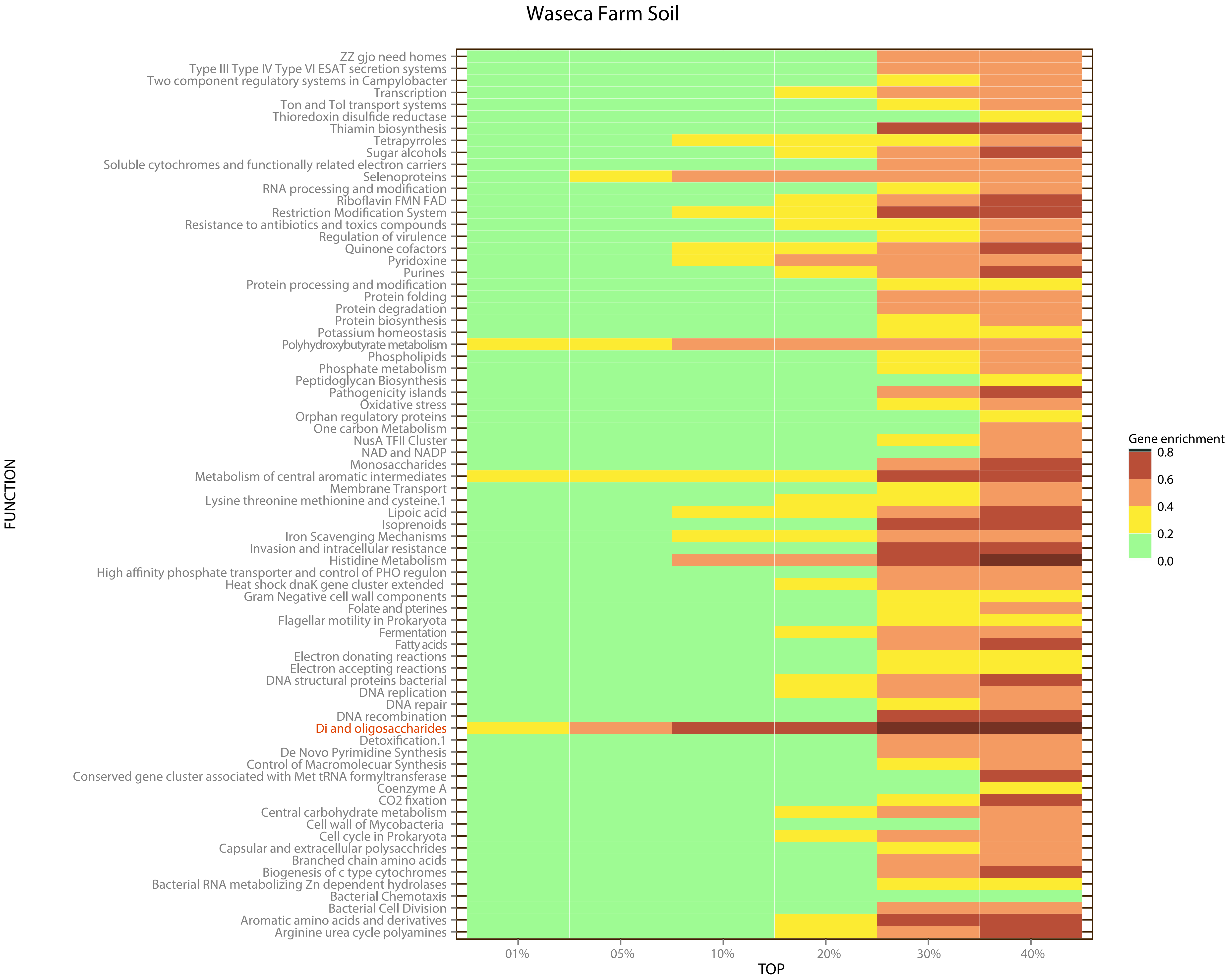

Supplementary figure 6. Results of the functional enrichment analysis for Waseca Farm Soil using the predefined bins (TOP: 1, 5, 10, 30, 40 %). Functions with a significant enrichment in the top 33% bin are shown in red.

Supplementary figure 7

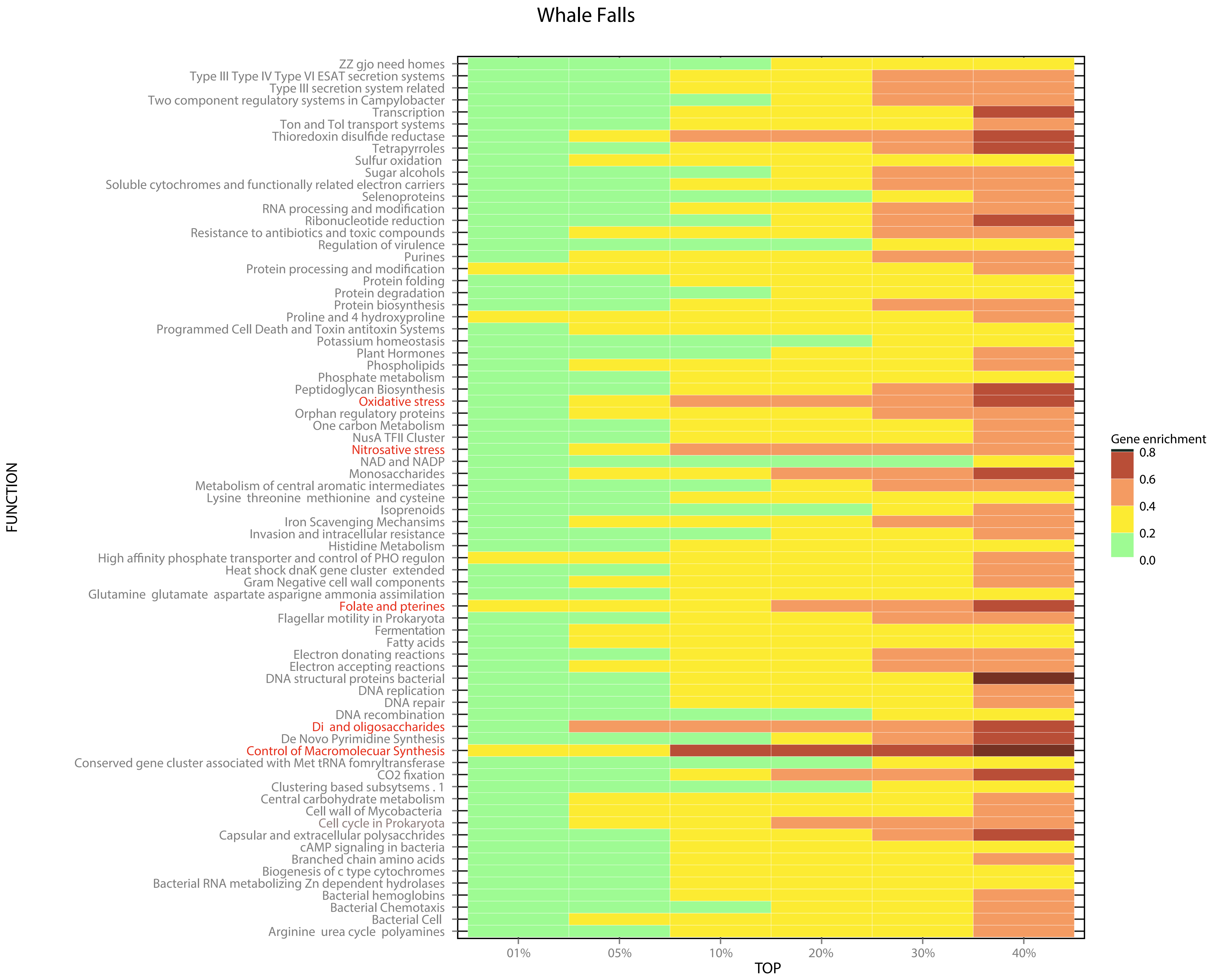

Supplementary figure 7. Results of the functional enrichment analysis for Whale Falls using the predefined bins (TOP: 1, 5, 10, 30, 40 %). Functions with a significant enrichment in the top 33% bin are shown in red.

Supplementary figure 8

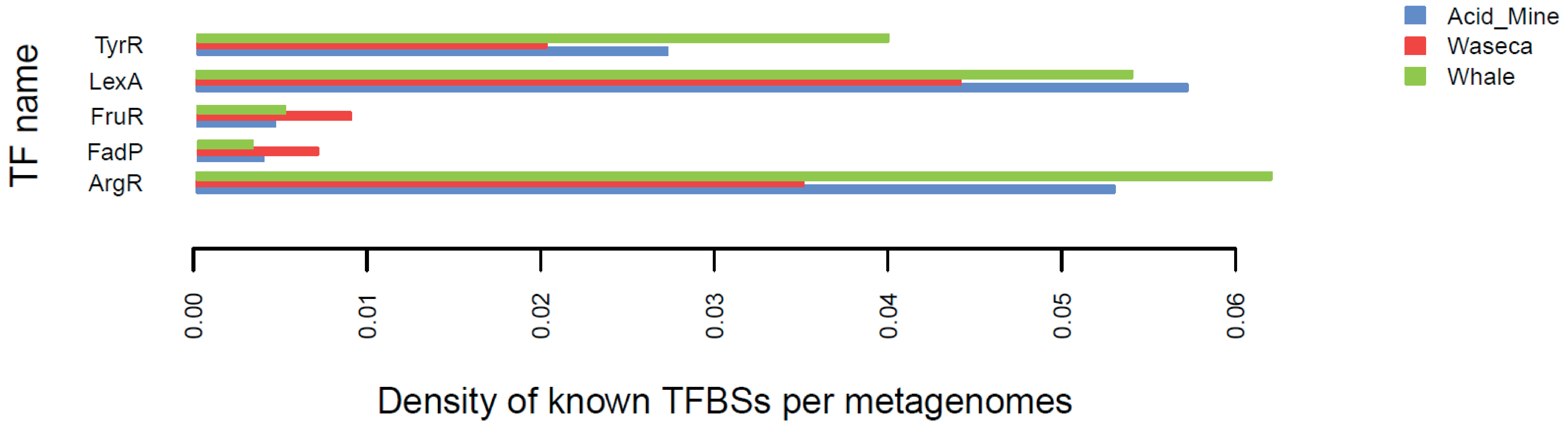

Supplementary figure 8. Comparison between relative abundances of our TFBS predictions that matched known TFBS found in the RegPrecise database. Only those known transcription factor binding sites whose presence is statistically different between environments are shown.
