# Supplementary material for: Adaptation to environmental factors shapes the organization of regulatory regions in microbial communities
Source: BMC Genomics. 2014 Oct 8;15(1):877. doi: 10.1186/1471-2164-15-877 (PMC4287501; doi:10.1186/1471-2164-15-877)
Supplement: Supplementary file 5 — Additional file 5: Table listing the number of TFBSs per genomes found after applying our method versus the number of sites described in Regprecise database. (PDF 88 KB) [file 12864_2014_6783_MOESM5_ESM.pdf]

Additional Material

Table 1. Number of transcription factor binding sites predicted per phylum in Acid Mine, Waseca Farm Soil and Whale Fall using the manually curated reconstructions of transcriptional regulons in RegPrecise Database.

AgaR

Phylum	Regulog	Genomes	Sites RegPrecise	Sites Metagenomic TFBS Searching
Proteobacteria	AgaR - Caulobacterales	4	1	0
Proteobacteria	AgaR - Pasteurellales	9	2	1
Proteobacteria	AgaR - Psychromonadaceae/Aeromonadales	6	11	0
Proteobacteria	AgaR - Xanthomonadales	4	2	0
Proteobacteria	AgaR2 - Enterobacteriales / Vibrionales	5	15	1
Proteobacteria	AgaR3 - Enterobacteriales / Vibrionales	6	7	0
Proteobacteria/gamma	AgaR - Enterobacteriales	12	15	3
Proteobacteria/gamma	AgaR - Shewanellaceae	16	10	0

AraR

Phylum	Regulog	Genomes	Sites RegPrecise	Sites Metagenomic TFBS Searching
Firmicutes	AraR - Bacillales	11	54	14
Firmicutes	AraR - Lactobacillales	16	19	5
Firmicutes	AraR1 - Clostridiaceae	20	12	5
Firmicutes	AraR2 - Clostridiaceae	20	11	4
Thermotogae	AraR - Thermotogales	11	35	8

ArgR

Phylum	Regulog	Genomes	Sites RegPrecise	Sites Metagenomic TFBS Searching
Firmicutes	ArgR - Bacillales	11	83	30
Firmicutes	ArgR - Staphylococcus	7	65	31
Proteobacteria/gamma	ArgR - Alteromonadales	9	61	34
Proteobacteria/gamma	ArgR - Enterobacteriales	12	259	113
Proteobacteria/gamma	ArgR - Pasteurellales	9	69	35
Proteobacteria/gamma	ArgR - Psychromonadaceae/Aeromonadales	6	70	34
Proteobacteria/gamma	ArgR - Shewanellaceae	16	502	177
Proteobacteria/gamma	ArgR - Vibrionales	10	107	43

BirA

Phylum	Regulog	Genomes	Sites RegPrecise	Sites Metagenomic TFBS Searching
Firmicutes	BirA - Bacillales	11	36	1
Firmicutes	BirA - Staphylococcus	7	21	16
Proteobacteria	BirA - Pseudomonadaceae	8	15	2
Proteobacteria/Gamma	BirA - Alteromonadales	9	17	4
Proteobacteria/Gamma	BirA - Oceanospirillales/Alteromonadales	12	26	2
Proteobacteria/Gamma	BirA - Psychromonadaceae/Aeromonadales	6	12	0
Proteobacteria/Gamma	BirA - Various betaproteobacteria	12	13	0
Proteobacteria/Gamma	BirA - Vibrionales	10	20	10
Proteobacteria/Gamma	BirA - Xanthomonadales	4	5	0
Proteobacteria/delta	BirA - Desulfovibrionales	13	8	6
Proteobacteria/delta	BirA - Desulfuromonadales	9	15	1
Proteobacteria/gamma	BirA - Enterobacteriales	12	24	2
Proteobacteria/gamma	BirA - Shewanellaceae	16	32	22

ExuR/UxuR

Phylum	Regulog	Genomes	Sites RegPrecise	Sites Metagenomic TFBS Searching
Proteobacteria	UxuR - Oceanospirillales/Alteromonadales	12	4	0
Proteobacteria	UxuR - Pasteurellales	9	19	5
Proteobacteria/alpha	UxuR - Rhodobacterales	15	5	0
Proteobacteria/gamma	ExuR - Enterobacteriales	12	47	8
Proteobacteria/gamma	UxuR - Enterobacteriales	12	42	4
Proteobacteria/gamma	UxuR - Psychromonadaceae/Aeromonadales	6	17	3
Proteobacteria/gamma	UxuR - Vibrionales	10	28	10

FabR

Phylum	Regulog	Genomes	Sites RegPrecise	Sites Metagenomic TFBS Searching
Proteobacteria/Gamma	FabR - Alteromonadales	9	45	0
Proteobacteria/Gamma	FabR - Oceanospirillales/Alteromonadales	12	8	0
Proteobacteria/Gamma	FabR - Psychromonadaceae/Aeromonadales	6	27	2
Proteobacteria/Gamma	FabR - Vibrionales	10	67	4
Proteobacteria/Gamma	FabR/DesT - Pseudomonadaceae	8	13	0

Phylum	Regulog	Genomes	Sites RegPrecise	Sites Metagenomic TFBS Searching
Proteobacteria/Gamma	FabR2 - Moraxellaceae	4	6	2
Proteobacteria/Gamma	FabR2 - Xanthomonadales	4	6	6
Proteobacteria/gamma	FabR - Enterobacteriales	12	40	1
Proteobacteria/gamma	FabR - Pasteurellales	9	19	1
Proteobacteria/gamma	FabR - Shewanellaceae	16	118	19

FadP

Phylum	Regulog	Genomes	Sites RegPrecise	Sites Metagenomic TFBS Searching
Proteobacteria/beta	FadP - Burkholderia	8	63	21
Proteobacteria/beta	FadP - Comamonadaceae	11	46	16
Proteobacteria/beta	FadP - Ralstonia	6	85	18

FadR

Phylum	Regulog	Genomes	Sites RegPrecise	Sites Metagenomic TFBS Searching
Proteobacteria	FadR - Pasteurellales	9	17	1
Proteobacteria	FadR - Vibrionales	10	59	33
Proteobacteria/gamma	FadR - Alteromonadales	9	34	24
Proteobacteria/gamma	FadR - Enterobacteriales	12	112	32
Proteobacteria/gamma	FadR - Psychromonadaceae/Aeromonadales	6	10	6
Proteobacteria/gamma	FadR - Shewanellaceae	16	78	49

FruR

Phylum	Regulog	Genomes	Sites RegPrecise	Sites Metagenomic TFBS Searching
Proteobacteria/gamma	FruR - Enterobacteriales	12	213	40
Proteobacteria/gamma	FruR - Oceanospirillales/Alteromonadales	12	4	1
Proteobacteria/gamma	FruR - Pasteurellales	9	2	0
Proteobacteria/gamma	FruR - Pseudomonadaceae	8	16	4
Proteobacteria/gamma	FruR - Psychromonadaceae/Aeromonadales	6	22	2
Proteobacteria/gamma	FruR - Vibrionales	10	60	16

HexR

Phylum	Regulog	Genomes	Sites RegPrecise	Sites Metagenomic TFBS Searching
Proteobacteria	HexR - Oceanospirillales/Alteromonadales	12	40	11
Proteobacteria	HexR1 - Pseudomonadaceae	8	30	0
Proteobacteria/beta	HexR - Burkholderia	8	38	0
Proteobacteria/beta	HexR - Comamonadaceae	11	28	6
Proteobacteria/beta	HexR - Ralstonia	6	24	0
Proteobacteria/beta	HexR - Various betaproteobacteria	12	12	0
Proteobacteria/gamma	HexR - Alteromonadales	9	38	20
Proteobacteria/gamma	HexR - Enterobacteriales	12	45	14
Proteobacteria/gamma	HexR - Pseudomonadaceae	8	44	1
Proteobacteria/gamma	HexR - Psychromonadaceae/Aeromonadales	6	79	22
Proteobacteria/gamma	HexR - Shewanellaceae	16	316	151
Proteobacteria/gamma	HexR - Vibrionales	10	190	64
Proteobacteria/gamma	HexR2 - Oceanospirillales/Alteromonadales	12	12	6

Irr

Phylum	Regulog	Genomes	Sites RegPrecise	Sites Metagenomic TFBS Searching
Proteobacteria/alpha	Irr - Rhizobiales	15	122	59
Proteobacteria/alpha	Irr - Rhodobacterales	15	38	13
Proteobacteria/alpha	Irr - Rhodospirillales	9	13	7

IscR

Phylum	Regulog	Genomes	Sites RegPrecise	Sites Metagenomic TFBS Searching
Proteobacteria	IscR - Betaproteobacteria	15	29	11
Proteobacteria/alpha	IscR - Rhodobacterales	10	91	29
Proteobacteria/gamma	IscR - Alteromonadales	18	86	33
Proteobacteria/gamma	IscR - Enterobacteriales	5	22	13
Proteobacteria/gamma	IscR - Pasteurellales	6	24	7
Proteobacteria/gamma	IscR - Pseudomonadales	4	6	1
Proteobacteria/gamma	IscR - Vibrionales	5	24	13

KdgR

Phylum	Regulog	Genomes	Sites RegPrecise	Sites Metagenomic TFBS Searching
Proteobacteria/gamma	KdgR - Enterobacteriales	12	155	36
Proteobacteria/gamma	KdgR - Pasteurellales	9	17	9
Proteobacteria/gamma	KdgR - Vibrionales	10	40	4
Thermotogae	KdgR - Thermotogales	11	26	8

LexA

Phylum	Regulog	Genomes	Sites RegPrecise	Sites Metagenomic TFBS Searching
Actinobacteria	LexA - Corynebacteriaceae	8	100	4
Chloroflexi	LexA - Chloroflexi	5	52	5
Cyanobacteria	LexA - Cyanobacteria	14	25	2
Firmicutes	LexA - Bacillales	11	202	14
Firmicutes	LexA - Staphylococcus	7	85	11
Proteobacteria	LexA - Desulfuromonadales	9	11	2
Proteobacteria	LexA - Rhodobacterales	15	128	2
Proteobacteria/Alphaproteobacteria	LexA - Caulobacterales	4	47	0
Proteobacteria/Alphaproteobacteria	LexA - Rhizobiales	15	212	15
Proteobacteria/Alphaproteobacteria	LexA - Rhodospirillales	9	52	2
Proteobacteria/Alphaproteobacteria	LexA - Sphingomonadales	7	61	1
Proteobacteria/Beta	LexA - Burkholderia	8	75	50
Proteobacteria/Beta	LexA - Comamonadaceae	11	71	49
Proteobacteria/Beta	LexA - Various betaproteobacteria	12	12	6
Proteobacteria/Gamma	LexA - Alteromonadales	9	111	64
Proteobacteria/Gamma	LexA - Oceanospirillales/Alteromonadales	12	91	64
Proteobacteria/Gamma	LexA - Pasteurellales	9	96	54
Proteobacteria/Gamma	LexA - Psychromonadaceae/Aeromonadales	6	77	39
Proteobacteria/Gamma	LexA - Xanthomonadales	4	6	0
Proteobacteria/Gamma	LexA2 - Pseudomonadaceae	9	5	0
Proteobacteria/Gamma	LexA2 - Xanthomonadales	4	2	0
Proteobacteria/beta	LexA - Ralstonia	6	62	34
Proteobacteria/delta	LexA - Desulfovibrionales	10	4	2
Proteobacteria/gamma	LexA - Enterobacteriales	12	253	159
Proteobacteria/gamma	LexA - Pseudomonadaceae	8	92	49
Proteobacteria/gamma	LexA - Shewanellaceae	16	192	103
Proteobacteria/gamma	LexA - Vibrionales	10	184	92

LiuQ

Phylum	Regulog	Genomes	Sites RegPrecise	Sites Metagenomic TFBS Searching
Proteobacteria/beta	LiuQ - Burkholderia	8	32	0
Proteobacteria/beta	LiuQ - Comamonadaceae	11	8	0
oteobacteria/beta	LiuQ - Ralstonia	6	14	6

LiuR

Phylum	Regulog	Genomes	Sites RegPrecise	Sites Metagenomic TFBS Searching
Proteobacteria	LiuR - Sphingomonadales	7	65	4
Proteobacteria/alpha	LiuR - Caulobacterales	4	14	2
Proteobacteria/alpha	LiuR - Rhizobiales	15	84	7
Proteobacteria/alpha	LiuR - Rhodobacterales	15	19	3
Proteobacteria/alpha	LiuR - Rhodospirillales	9	17	1
Proteobacteria/beta	LiuR - Burkholderia	8	6	1
Proteobacteria/beta	LiuR - Comamonadaceae	11	59	0
Proteobacteria/beta	LiuR - Ralstonia	6	57	3
Proteobacteria/beta	LiuR - Various betaproteobacteria	12	31	8
Proteobacteria/gamma	LiuR - Alteromonadales	9	68	15
Proteobacteria/gamma	LiuR - Oceanospirillales/Alteromonadales	12	28	2
Proteobacteria/gamma	LiuR - Pseudomonadaceae	8	32	9
Proteobacteria/gamma	LiuR - Psychromonadaceae/Aeromonadales	6	20	4
Proteobacteria/gamma	LiuR - Shewanellaceae	16	134	11
Proteobacteria/gamma	LiuR - Vibrionales	10	38	12

ModE

Phylum	Regulog	Genomes	Sites RegPrecise	Sites Metagenomic TFBS Searching
Proteobacteria/Alpha	ModE - Caulobacterales	4	5	1
Proteobacteria/Alpha	ModE - Rhizobiales	15	11	6
Proteobacteria/Beta	ModE - Burkholderia	8	8	8
Proteobacteria/Beta	ModE - Comamonadaceae	11	10	3
Proteobacteria/Delta	ModE - Desulfuromonadales	9	10	9
Proteobacteria/Gamma	ModE - Moraxellaceae	4	1	0
Proteobacteria/Gamma	ModE - Oceanospirillales/Alteromonadales	12	2	2
Proteobacteria/alpha	ModE - Rhodobacterales	15	5	2
Proteobacteria/alpha	ModE - Rhodospirillales	9	2	2
Proteobacteria/alpha	ModE - Sphingomonadales	7	2	2
Proteobacteria/beta	ModE - Ralstonia	6	8	5
Proteobacteria/beta	ModE - Various betaproteobacteria	12	5	3
Proteobacteria/delta	ModR - Desulfovibrionales	13	25	15
Proteobacteria/gamma	ModE - Enterobacteriales	12	55	47
Proteobacteria/gamma	ModE - Pasteurellales	9	21	20
Proteobacteria/gamma	ModE - Pseudomonadaceae	8	9	9
Proteobacteria/gamma	ModE - Psychromonadaceae/Aeromonadales	6	3	3
Proteobacteria/gamma	ModE - Shewanellaceae	16	10	10
Proteobacteria/gamma	ModE - Vibrionales	10	2	2

NadQ

Phylum	Regulog	Genomes	Sites RegPrecise	Sites Metagenomic TFBS Searching
Proteobacteria/Alpha	NadQ - Caulobacterales	4	16	9
Proteobacteria/Alpha	NadQ - Rhizobiales	15	22	7
Proteobacteria/Alpha	NadQ - Rhodobacterales	15	5	2
Proteobacteria/Alpha	NadQ - Rhodospirillales	9	10	4
Proteobacteria/Beta	NadQ - Comamonadaceae	11	6	4
Proteobacteria/Beta	NadQ - Various betaproteobacteria	12	2	2
Proteobacteria/Gamma	NadQ - Moraxellaceae	4	6	5

NagC

Phylum	Regulog	Genomes	Sites RegPrecise	Sites Metagenomic TFBS Searching
Proteobacteria/gamma	NagC - Enterobacteriales	12	68	42
Proteobacteria/gamma	NagC - Oceanospirillales/Alteromonadales	12	5	4
Proteobacteria/gamma	NagC - Pasteurellales	9	16	7
Proteobacteria/gamma	NagC - Psychromonadaceae/Aeromonadales	6	16	9
Proteobacteria/gamma	NagC - Vibrionales	10	199	111

NagQ

Phylum	Regulog	Genomes	Sites RegPrecise	Sites Metagenomic TFBS Searching
Proteobacteria/alpha	NagQ - Caulobacterales	4	13	0
Proteobacteria/alpha	NagQ - Rhizobiales	15	16	0
Proteobacteria/alpha	NagQ - Rhodobacterales	15	8	0
Proteobacteria/alpha	NagQ - Rhodospirillales	9	6	0
Proteobacteria/beta	NagQ - Burkholderia	8	7	0
Proteobacteria/beta	NagQ - Ralstonia	6	2	0
Proteobacteria/beta	NagQ - Various betaproteobacteria	12	12	4
Proteobacteria/gamma	NagQ - Oceanospirillales/Alteromonadales	12	11	2
Proteobacteria/gamma	NagQ - Pseudomonadaceae	8	4	0
Proteobacteria/gamma	NagQ - Xanthomonadales	4	2	0

NagR

Phylum	Regulog	Genomes	Sites RegPrecise	Sites Metagenomic TFBS Searching
Proteobacteria/gamma	NagR - Alteromonadales	9	27	4
Proteobacteria/gamma	NagR - Oceanospirillales/Alteromonadales	12	19	1
Proteobacteria/gamma	NagR - Shewanellaceae	16	105	0
Proteobacteria/gamma	NagR - Xanthomonadales	4	17	0

NiaR

Phylum	Regulog	Genomes	Sites RegPrecise	Sites Metagenomic TFBS Searching
Firmicutes	NiaR - Bacillales	11	26	15
Firmicutes	NiaR - Clostridiaceae	20	27	9
Firmicutes	NiaR - Lactobacillaceae	15	7	0
Firmicutes	NiaR - Streptococcaceae	15	23	8
Thermotogae	NiaR - Thermotogales	11	13	0

NikR

Phylum	Regulog	Genomes	Sites RegPrecise	Sites Metagenomic TFBS Searching
Proteobacteria/alpha	NikR - Rhizobiales	15	11	5
Proteobacteria/alpha	NikR - Rhodobacterales	15	2	1
Proteobacteria/alpha	NikR - Rhodospirillales	9	2	0
Proteobacteria/beta	NikR - Burkholderia	8	2	0
Proteobacteria/beta	NikR - Comamonadaceae	11	3	1
Proteobacteria/beta	NikR - Various betaproteobacteria	12	2	0
Proteobacteria/delta	NikR - Desulfovibrionales	13	19	7
Proteobacteria/delta	NikR - Desulfuromonadales	9	7	0
Proteobacteria/gamma	NikR - Enterobacteriales	12	9	6
Proteobacteria/gamma	NikR - Pseudomonadaceae	8	2	0
Proteobacteria/gamma	NikR - Shewanellaceae	16	8	8

NorR

Phylum	Regulog	Genomes	Sites RegPrecise	Sites Metagenomic TFBS Searching
Proteobacteria/beta	NorR - Burkholderia	8	5	3
Proteobacteria/beta	NorR - Comamonadaceae	11	25	6
Proteobacteria/beta	NorR - Ralstonia	6	35	8
Proteobacteria/gamma	NorR - Alteromonadales	9	18	4
Proteobacteria/gamma	NorR - Enterobacteriales	12	33	28
Proteobacteria/gamma	NorR - Oceanospirillales/Alteromonadales	12	13	5
Proteobacteria/gamma	NorR - Pseudomonadaceae	8	40	6
Proteobacteria/gamma	NorR - Psychromonadaceae/Aeromonadales	6	21	4
Proteobacteria/gamma	NorR - Shewanellaceae	16	91	21
Proteobacteria/gamma	NorR - Vibrionales	10	40	15
Proteobacteria/gamma	NorR2 - Vibrionales	10	19	3

NrdR

Phylum	Regulog	Genomes	Sites RegPrecise	Sites Metagenomic TFBS Searching
Actinobacteria	NrdR - Actinobacteria	15	57	10
Chlamydiae	NrdR - Chlamydia	5	10	2
Cyanobacteria	NrdR - Cyanobacteria	5	9	1
Deinococcus-Thermus	NrdR - Deinococcus-Thermus	2	5	3
Firmicutes	NrdR - Firmicutes	33	167	56
Proteobacteria	NrdR - Alphaproteobacteria	8	27	5
Proteobacteria	NrdR - Betaproteobacteria	9	20	5
Proteobacteria	NrdR - Deltaproteobacteria	6	18	6
Proteobacteria	NrdR - Gammaproteobacteria	35	151	28
Thermotogae	NrdR - Thermotogales	11	60	29
unclassified	NrdR - Mixture	5	11	3

NrtR

Phylum	Regulog	Genomes	Sites RegPrecise	Sites Metagenomic TFBS Searching
Actinobacteria	NrtR - Actinobacteria-1	6	9	5
Actinobacteria	NrtR - Actinobacteria-2	5	5	1
Actinobacteria	NrtR - Actinobacteria-3	3	5	1
Actinobacteria	NrtR - Actinobacteria-4	5	11	4
Bacteroidetes	NrtR - Bacteroidetes-1	2	2	0
Bacteroidetes	NrtR - Bacteroidetes-2	4	15	5
Bacteroidetes	NrtR - Bacteroidetes-3	3	9	3
Bacteroidetes	NrtR - Cytophaga	2	4	4
Chloroflexi	NrtR - Chloroflexi	1	4	1
Cyanobacteria	NrtR - Cyanobacteria	7	14	4
Firmicutes	NrtR - Firmicutes-1	4	6	1
Firmicutes	NrtR - Firmicutes-2	3	3	0
Firmicutes	NrtR - Firmicutes-3	1	3	2
Firmicutes	NrtR - Firmicutes-4	1	2	2
Planctomycetes	NrtR - Pirellula	1	2	0
Proteobacteria	NrtR - Gammaproteobacteria-1	6	14	4
Proteobacteria	NrtR - Gammaproteobacteria-2	3	5	1
Proteobacteria	NrtR - Gammaproteobacteria-3	3	4	3

NsrR

Phylum	Regulog	Genomes	Sites RegPrecise	Sites Metagenomic TFBS Searching
Firmicutes	NsrR - Bacillales	11	30	17
Proteobacteria/alpha	NsrR - Caulobacterales	4	3	2
Proteobacteria/alpha	NsrR - Rhizobiales	15	2	0
Proteobacteria/alpha	NsrR - Rhodobacterales	15	17	4
Proteobacteria/alpha	NsrR - Rhodospirillales	9	13	5
Proteobacteria/alpha	NsrR - Sphingomonadales	7	5	4
Proteobacteria/beta	NsrR - Burkholderia	8	23	12
Proteobacteria/beta	NsrR - Comamonadaceae	11	15	10
Proteobacteria/beta	NsrR - Neisseriales	12	5	4
Proteobacteria/beta	NsrR - Ralstonia	6	23	18
Proteobacteria/beta	NsrR - Various betaproteobacteria	12	16	9
Proteobacteria/gamma	NsrR - Alteromonadales	9	2	1
Proteobacteria/gamma	NsrR - Enterobacteriales	12	73	39
Proteobacteria/gamma	NsrR - Moraxellaceae	4	7	0
Proteobacteria/gamma	NsrR - Oceanospirillales/Alteromonadales	12	8	6
Proteobacteria/gamma	NsrR - Psychromonadaceae/Aeromonadales	6	5	2
Proteobacteria/gamma	NsrR - Shewanellaceae	16	51	22
Proteobacteria/gamma	NsrR - Vibrionales	10	40	14

PdhR

Phylum	Regulog	Genomes	Sites RegPrecise	Sites Metagenomic TFBS Searching
Proteobacteria/gamma	PdhR - Alteromonadales	9	15	6
Proteobacteria/gamma	PdhR - Enterobacteriales	12	36	25
Proteobacteria/gamma	PdhR - Oceanospirillales/Alteromonadales	12	22	12
Proteobacteria/gamma	PdhR - Psychromonadaceae/Aeromonadales	6	8	6
Proteobacteria/gamma	PdhR - Shewanellaceae	16	109	37
Proteobacteria/gamma	PdhR - Vibrionales	10	16	12

PsrA

Phylum	Regulog	Genomes	Sites RegPrecise	Sites Metagenomic TFBS Searching
Proteobacteria	PsrA - Xanthomonadales	4	7	5
Proteobacteria/alpha	PsrA - Caulobacterales	4	70	28
Proteobacteria/alpha	PsrA - Rhizobiales	15	12	6
Proteobacteria/beta	PsrA - Burkholderia	8	16	9
Proteobacteria/beta	PsrA - Ralstonia	6	9	4
Proteobacteria/beta	PsrA - Various betaproteobacteria	12	34	8
Proteobacteria/gamma	PsrA - Alteromonadales	9	26	4
Proteobacteria/gamma	PsrA - Oceanospirillales/Alteromonadales	12	67	21
Proteobacteria/gamma	PsrA - Pseudomonadaceae	8	69	18
Proteobacteria/gamma	PsrA - Psychromonadaceae/Aeromonadales	6	19	4
Proteobacteria/gamma	PsrA - Shewanellaceae	16	295	105
Proteobacteria/gamma	PsrA - Vibrionales	10	49	15

PurR

Phylum	Regulog	Genomes	Sites RegPrecise	Sites Metagenomic TFBS Searching
Proteobacteria/gamma	PurR - Enterobacteriales	12	298	71
Proteobacteria/gamma	PurR - Pasteurellales	9	77	14
Proteobacteria/gamma	PurR - Vibrionales	10	149	29

RbsR

Phylum	Regulog	Genomes	Sites RegPrecise	Sites Metagenomic TFBS Searching
Firmicutes	RbsR - Bacillales	11	12	11
Firmicutes	RbsR - Staphylococcus	7	2	2
Firmicutes	RbsR - Streptococcus	8	4	1
Firmicutes	RbsR2 - Staphylococcus	7	4	4
Proteobacteria/beta	RbsR - Burkholderia	8	7	0
Proteobacteria/beta	RbsR - Comamonadaceae	11	2	0
Proteobacteria/beta	RbsR - Desulfovibrionales	10	1	0
Proteobacteria/beta	RbsR - Ralstonia	6	2	0
Proteobacteria/gamma	RbsR - Enterobacteriales	12	17	1
Proteobacteria/gamma	RbsR - Pasteurellales	9	6	0
Proteobacteria/gamma	RbsR - Pseudomonadaceae	8	5	0
Proteobacteria/gamma	RbsR - Psychromonadaceae/Aeromonadales	6	5	0
Proteobacteria/gamma	RbsR - Shewanellaceae	16	4	0
Proteobacteria/gamma	RbsR - Vibrionales	10	15	0
Thermotogae	RbsR - Thermotogales	11	15	3

Rex

Phylum	Regulog	Genomes	Sites RegPrecise	Sites Metagenomic TFBS Searching
Actinobacteria	Rex - Actinomycetales	18	88	4
Chloroflexi	Rex - Chloroflexi	5	39	5
Deinococcus-Thermus	Rex - Deinococcus-Thermus	5	10	0
Firmicutes	Rex - Bacillales	11	62	26
Firmicutes	Rex - Clostridiaceae	20	191	122
Firmicutes	Rex - Lactobacillaceae	15	70	24
Firmicutes	Rex - Staphylococcus	7	74	18
Firmicutes	Rex - Streptococcaceae	15	160	52
Firmicutes	Rex - Thermoanaerobacterales	3	42	29
Proteobacteria/delta	Rex - Desulfovibrionales	10	100	37
Thermotogae	Rex - Thermotogales	11	114	41
Thermotogae	Rex1 - Thermotogales	11	10	2

RutR

Phylum	Regulog	Genomes	Sites RegPrecise	Sites Metagenomic TFBS Searching
Proteobacteria/Alpha	RutR - Caulobacterales	4	4	0
Proteobacteria/Alpha	RutR - Rhizobiales	15	44	12
Proteobacteria/Alpha	RutR - Rhodobacterales	15	62	19
Proteobacteria/Alpha	RutR - Rhodospirillales	9	2	0
Proteobacteria/Beta	RutR - Burkholderia	8	12	2
Proteobacteria/Beta	RutR - Ralstonia	6	4	0
Proteobacteria/Gamma	RutR - Alteromonadales	9	19	5
Proteobacteria/Gamma	RutR - Moraxellaceae	4	8	4
Proteobacteria/Gamma	RutR - Oceanospirillales/Alteromonadales	12	32	6
Proteobacteria/Gamma	RutR - Pseudomonadales	8	29	13
Proteobacteria/Gamma	RutR2 - Pseudomonadales	8	7	3
Proteobacteria/Gamma	RutR3 - Pseudomonadales	8	23	13
Proteobacteria/Gamma	RutR3 - Psychromonadaceae/Aeromonadales	6	4	0
Proteobacteria/Gamma	RutR3 - Vibrionales	10	4	2
Proteobacteria/Gamma	RutR4 - Alteromonadales	9	2	2
Proteobacteria/Gamma	RutR4 - Pseudomonadales	9	4	0
Proteobacteria/gamma	RutR - Enterobacteriales	12	13	0

TrpR

Phylum	Regulog	Genomes	Sites RegPrecise	Sites Metagenomic TFBS Searching
Proteobacteria/gamma	TrpR - Enterobacteriales	12	44	4
Proteobacteria/gamma	TrpR - Moraxellaceae	4	3	3
Proteobacteria/gamma	TrpR - Oceanospirillales/Alteromonadales	12	3	1
Proteobacteria/gamma	TrpR - Pasteurellales	9	24	8
Proteobacteria/gamma	TrpR - Psychromonadaceae/Aeromonadales	6	2	0
Proteobacteria/gamma	TrpR - Shewanellaceae	16	30	8
Proteobacteria/gamma	TrpR - Vibrionales	10	25	5
Proteobacteria/gamma	TrpR - Xanthomonadales	4	3	2

TyrR

Phylum	Regulog	Genomes	Sites RegPrecise	Sites Metagenomic TFBS Searching
Proteobacteria/gamma	PhhR - Pseudomonadales	8	32	19
Proteobacteria/gamma	TyrR - Alteromonadales	9	42	20
Proteobacteria/gamma	TyrR - Enterobacteriales	12	107	57
Proteobacteria/gamma	TyrR - Pasteurellales	9	29	13
Proteobacteria/gamma	TyrR - Psychromonadaceae/Aeromonadales	6	17	11
Proteobacteria/gamma	TyrR - Shewanellaceae	16	309	135
Proteobacteria/gamma	TyrR - Vibrionales	12	67	43

XylR

Phylum	Regulog	Genomes	Sites RegPrecise	Sites Metagenomic TFBS Searching
Firmicutes	XylR - Bacillales	11	41	32
Firmicutes	XylR - Enterococcaceae	2	4	2
Firmicutes	XylR - Lactobacillaceae	15	15	9
Firmicutes	XylR1 - Clostridiaceae	20	22	16
Firmicutes	XylR2 - Clostridiaceae	20	9	5
Proteobacteria/alpha	XylR - Rhizobiales	15	19	18

Zur

Phylum	Regulog	Genomes	Sites RegPrecise	Sites Metagenomic TFBS Searching
Actinobacteria	zur - Actinobacteria	9	23	22
Cyanobacteria	zur - Cyanobacteria	9	15	13
Firmicutes	zur - Bacilli	14	69	43
Firmicutes	zur - Clostridia	6	12	7
Proteobacteria	zur - Alphaproteobacteria-1	8	19	12
Proteobacteria	zur - Alphaproteobacteria-2	4	4	2
Proteobacteria	zur - Alphaproteobacteria-3	12	19	17
Proteobacteria	zur - Alphaproteobacteria-4	8	17	9
Proteobacteria	zur - Betaproteobacteria-1	6	18	9
Proteobacteria	zur - Gammaproteobacteria	34	118	69
Proteobacteria	zur - Proteobacteria	11	21	12
Thermotogae	zur - Thermotoga	5	6	4
